# Association of DRD4 exon III and 5-HTTLPR VNTR genetic polymorphisms with psychiatric symptoms in hemodialysis patients

**DOI:** 10.1371/journal.pone.0249284

**Published:** 2021-03-30

**Authors:** Osama Y. Alshogran, Laith N. Al-Eitan, Shoroq M. Altawalbeh, Hatem A. Aman

**Affiliations:** 1 Department of Clinical Pharmacy, Faculty of Pharmacy, Jordan University of Science and Technology, Irbid, Jordan; 2 Department of Applied Biological Sciences, Jordan University of Science and Technology, Irbid, Jordan; 3 Department of Biotechnology and Genetic Engineering, Jordan University of Science and Technology, Irbid, Jordan; National Institutes of Health, UNITED STATES

## Abstract

Mental illness is prevalent among hemodialysis (HD) patients. Given that the dopaminergic and serotonergic pathways are involved in the etiology of psychiatric disease, this study evaluated the genetic association of dopamine D4 receptor (*DRD4*) and serotonin transporter (*SLC6A4*) genes with psychiatric symptom susceptibility among HD patients. Hospital Anxiety and Depression Scale (HADS) was used to assess anxiety and depressive symptoms among patients (*n* = 265). Genetic polymorphisms of *DRD4* (48 bp VNTR) and *SLC6A4* (5-HTTLPR VNTR and rs25531) were examined using a conventional polymerase chain reaction-restriction fragment length polymorphism (PCR-RFLP) technique, as appropriate. Significant differences were observed in the distribution of 5-HTTLPR genotypes, *SLC6A4* tri-allelic-phased genotype, and *DRD4*-Exon III VNTR genotypes/alleles between patients with anxiety symptoms versus those with normal/borderline conditions (*p*<0.05). Binary logistic regression analyses showed that the heterozygous 4,5 VNTR genotype of *DRD4* was associated with a higher risk of anxiety symptoms after adjusting for other covariates (odds ratio = 4.25, *p* = 0.028). None of the studied polymorphisms was linked to depression in HD patients. Collectively, the current findings provide genetic clues to psychopathology in HD patients and suggest that the *DRD4* exon III VNTR polymorphism is involved in the etiology of anxiety in this patient population.

## Introduction

Psychiatric symptoms such as anxiety and depression are common among patients undergoing hemodialysis (HD) [1]. These symptoms pose a threat to human health, as they are associated with adverse clinical outcomes such as increased hospitalization and mortality [2, 3]. Multiple factors could be associated with vulnerability to psychiatric symptoms, including sociodemographic, clinical, and environmental elements, lifestyle, and the genetic background [4–6]. For instance, one study suggested 151 genes that could potentially be involved in major depressive disorder [7]. The dopaminergic and serotonergic pathways are particularly promising avenues of investigation because of their critical involvement in the pathophysiology of mental health, and they could thus be significant determinants of psychiatric symptoms among patient populations [8, 9].

To date, only a small number of studies have examined the genetic contribution to psychiatric disease in HD patients [4, 10]. For instance, it has been previously shown that the neuropeptide S receptor1 (NPSR1) Asn107Ile polymorphism was associated with anxiety in HD patients [4]. The dopamine receptor D4 (*DRD4*) gene 48 bp variable number of tandem repeats (VNTR) polymorphism at the third exon has been previously reported to be linked with mental illness or behavioral traits in different patient settings [11, 12]. For example, the long version of the polymorphism (7–11 repeat) was associated with severe substance abuse problems [13]. Other studies have also correlated *DRD4* variant repeat alleles with depressive symptoms in different populations such as adulthood [14]. Variants of *DRD4* could also modulate the response to treatment in patients with depression [15]. Furthermore, *DRD4* haplotypes may contribute to psychopathological features, including anxiety in subjects with eating disorders [16]. To date, the association of the *DRD4* repeat variant with psychiatric symptoms in HD patients has not been clarified.

Serotonergic neurotransmission contributes to the etiology of psychiatric illnesses such as depression [17]. Serotonin transporter (5-HTT) regulates serotonin reuptake into the presynaptic neuron and the distribution of serotonin inside and outside the cell. Polymorphisms in the serotonin transporter gene (*SLC6A4*), such as the 5-HTTLPR (LL/LS/SS) variant and the rs25531 (A/G) marker located at the upstream regulatory region, have garnered interest because they can influence the transcriptional activity of the gene [18]. The 5-HTTLPR polymorphism comprises the short (*S*) deletion allele and the long (*L*) insertion allele [19]. The 5-HTTLPR variants have been linked to depression in patients with coronary heart disease [20], Parkinson’s disease [21], or postpartum depression [22], as well as anxiety-related personality measures [23]. To our knowledge, information about the possible association between the genetic variants 5-HTTLPR and rs25531 and psychiatric symptoms among HD patients is scarce [10].

Thus, the objective of this study was to examine the association of *DRD4* and *SLC6A4* genetic polymorphisms with depression or anxiety in HD patients in Jordan.

## Methods

### Study sample and setting

This study included HD patients from six primary dialysis centers located in governmental, military, and educational hospitals in Jordan. Subjects included were those adults who had been undergoing chronic HD for a period of three months or more. Patients receiving antidepressants or anxiety medications were excluded. Eligible subjects were invited to participate at the scheduled dialysis sessions. Written informed consent was obtained from all study participants. The study protocol was approved by the institutional review board committees at Jordan University of Science and Technology (No. 26/102/2017), Ministry of Health (No. 3206), Jordan University Hospital (No. 10/2017/18685) and Royal Medical Services.

### Genotyping analysis

Peripheral blood samples (5 mL) were collected from patients into an EDTA-containing tube. Genomic DNA was then extracted from blood using QIAamp DNA Mini Kit according to the manufacturer’s instructions (Qiagen, Germany). The yield (ng/μl) and quality (A260/280) of DNA were assessed using a NanoDrop ND-1000 spectrophotometer (Thermo Fisher Scientific Inc., DE, USA). DNA samples were subsequently stored at -20°C until further use.

The desired DNA target of the *DRD4* 48 bp tandem repeats polymorphism was amplified using Polymerase Chain Reaction (PCR) through specific primers, as described earlier [24]. PCR amplification was conducted in a total reaction volume of 24 μL containing master mix (Solis BioDyne, Germany), 1.5 μL of each primer, 4 μL genomic DNA, and nuclease-free water. The PCR products were electrophoresed on 2% agarose gel and visualized by ethidium bromide staining. Images of the gel were also obtained using the Molecular Imager ChemiDoc™ XRS system (Bio-Rad Laboratories, California, USA).

The 5-HTTLPR variants and the SNP at rs25531 were analyzed in two stages, as previously described [25]. The insertion (*L* allele) or the absence of the insertion (*S* allele) of 43 bp of 5-HTTLPR variants were determined after PCR analysis of the genomic DNA. The PCR reaction for *SLC6A4* was conducted in a total volume of 24 μL containing master mix (Solis BioDyne, Germany), 1.5 μL of each primer, 4 μL genomic DNA, and nuclease-free water. Products were subsequently separated on 2% gel electrophoresis and visualized by ethidium bromide staining. The large product size (512 bp) of PCR represents the *L* allele, while the small product size (469 bp) represents the *S* allele. Restriction Fragment Length Polymorphism (RFLP)–PCR was then conducted to identify rs25531 alleles. In brief, the PCR product (10 μL) was digested by adding 0.3 μL of *Msp1* (20 U/ml; New England Biolabs, USA) at 37°C for 6 h. The digested products were loaded and separated using 3% agarose gel and then visualized as above. The G allele of rs25531 will yield a product of 174 bp.

### Measures

#### Dependent variables

Depressive and anxiety symptoms were used as dependent variables. These symptoms were assessed using the Hospital Anxiety and Depression Scale (HADS), which was validated and frequently used with dialysis patients as well as in Arabic speaking countries. It is a concise and easy-to-complete self-assessment instrument that contains 14 statements measuring symptoms of depression (HADS-D) and anxiety (HADS-A) over the preceding week. Patients were asked to rate 7 items for each subscale, and each response was rated on a four-point Likert scale (0 to 3). Responses were summed so that the total score of each subscale ranged between 0 and 21, with higher scores reflecting the severity of the symptoms. The scores were classified as normal-borderline (HADS-A or HADS-D < 11) or cases (HADS-A or HADS-D ≥ 11) [5]. The research questionnaire was initially piloted among groups of dialysis patients to ensure the clarity and understandability of the statements. The Cronbach alpha of the current study was 0.84 for both HADS-D and HADS-A.

#### Independent variables

Primary independent predictors were the *DRD4* and *SLC6A4* polymorphisms. Data for other variables were also collected, including 1) demographics, such as age and gender; 2) socioeconomic information, such as marital status, smoking habits, and income; 3) laboratory values, including serum creatinine and urea; 4) dialysis-related factors, such as length of dialysis session, time under dialysis, and number of sessions per week; and 5) medical factors, such as disease history. Data were obtained from the patients or medical records as appropriate.

### Statistical analyses

The demographics and clinical characteristics of study subjects were presented using descriptive statistics (mean, standard deviation, frequency, and percentages), as appropriate. Differences in demographic and clinical parameters between patient groups were analyzed using students t-test for continuous variables and Chi-square test for categorical data. The genotype and allele frequencies of *DRD4* and *SLC6A4* polymorphisms were compared between groups using Chi-square test or Fisher exact test when appropriate.

Binary logistic regression analysis was performed to identify the possible contribution of genetic polymorphisms on depression and anxiety after adjustment for other covariates. Clinical and demographic co-variables were selected by backward stepwise process, with *p* < 0.2 to stay. The associations were expressed as odds ratios (ORs) and were considered statistically significant when *p* < 0.05. Statistical analyses were conducted using STATA version 14 (StataCorp, 2015, College Station, TX, USA).

## Results

### Participant characteristics

The current study included 265 HD patients. The mean age (±SD) of the sample was 52.6 ± 16.1 years, and about 59% were males. The majority were married (72.4%) and nonsmokers (80.8%). Obesity was present in 19.2% of participants, while hypertension and diabetes were more frequent, with prevalence of 71% and 36.6%, respectively. The majority of patients received dialysis three times a week (78.9%), and the length of dialysis sessions ranged from 3 to 4.5 hours. The mean depression score (HADS-D) was 8.31 ± 5.2, and 34.7% were defined as cases, while the average anxiety score (HADS-A) was 7.15 ± 4.9, and 27.2% were defined as cases.

### Demographic and clinical factors based on depression and anxiety symptoms

Various factors were significantly different between depressive cases and controls, such as age, education level, hypertension and diabetes prevalence, and dialysis sessions per week, as listed in Table 1. For instance, hypertension was more frequent in depressive cases (81.5%) than in patients without depression (65.3%, *p* = 0.006). Furthermore, marital status, education level, hypertension, diabetes, number of dialysis years, dialysis sessions per week, and urea levels were different between anxiety cases and controls. For example, HD patients with anxiety tended to have lower levels of higher education as well as more hypertension and diabetes (*p* < 0.05) compared to patients without anxiety symptoms, as shown in Table 1.

**Table 1:** Demographics, socioeconomics and clinical characteristics of patients by depression and anxiety status.

	Depression (HADS-D)			Anxiety (HADS-A)		
	Normal-borderline (173)	Case (92)	P	Normal-borderline (193)	Case (72)	P
Age, years (mean+SD)	51±16.4	55.7±15.1	**0.023**	51.8±6.2	54.9±15.5	0.158
Gender (n, %)			0.237			0.304
Female	66 (38.2)	42 (45.6)		75 (38.9)	33 (45.8)	
Male	107 (61.8)	50 (54.4)		118 (61.1)	39 (54.2)	
Marital status (n, %)			0.138			**0.013**
Single	47 (27.2)	15 (16.3)		53 (27.5)	9 (12.5)	
Married	118 (68.2)	74 (80.4)		130 (67.4)	62 (86.1)	
Divorced	3 (1.7)	2 (2.2)		4 (2.1)	1 (1.4)	
Widowed	5 (2.9)	1 (1.1)		6 (3.1)	0 (0)	
Education level (n, %)			**<0.0001**			**0.001**
Illiterate	19 (11.2)	27 (30)		24 (12.6)	22 (31.4)	
Junior school	40 (23.5)	26 (28.9)		47 (24.7)	19 (27.1)	
High school	60 (35.3)	23 (25.6)		63 (33.2)	20 (28.7)	
College or over	51 (30)	14 (15.5)		56 (29.5)	9 (12.8)	
Body mass index (kg/m^2^) (n, %)			0.121			0.158
<18.5	11 (6.4)	4 (4.4)		12 (6.2)	3 (4.2)	
18.5–24.9	85 (49.1)	33 (35.9)		93 (48.2)	25 (34.7)	
25–29.9	46 (26.6)	35 (38)		55 (28.5)	26 (36.1)	
>30	31 (17.9)	20 (21.7)		33 (17.1)	18 (25)	
Smoking (n, %)			0.124			0.764
No	135 (78)	79 (85.9)		155 (80.3)	59 (81.9)	
Yes	38 (22)	13 (14.1)		38 (19.7)	13 (18.1)	
Hypertension (n, %)			**0.006**			**0.035**
No	60 (34.7)	17 (18.5)		63 (32.6)	14 (19.4)	
Yes	113 (65.3)	75 (81.5)		130 (67.4)	58 (80.6)	
Diabetes mellitus (n, %)			**0.05**			**0.028**
No	117 (67.6)	51 (55.4)		130 (67.4)	38 (52.8)	
Yes	56 (32.4)	41 (44.6)		63 (32.6)	34 (47.2)	
Income (n, %)			0.558			0.089
<250 JD	61 (35.9)	31 (33.7)		74 (38.7)	18 (24.4)	
250–500 JD	86 (50.6)	52 (56.5)		93 (48.7)	45 (63.4)	
>500 JD	23 (13.5)	9 (9.8)		24 (12.6)	8 (11.2)	
Number of years under dialysis (n, %)			0.235			**0.01**
<2 years	44 (25.4)	19 (20.7)		46 (23.8)	17 (23.6)	
2–5 years	57 (33)	40 (43.5)		61 (31.6)	36 (50)	
>5 years	72 (41.6)	33 (35.8)		86 (44.6)	19 (26.4)	
Dialysis sessions per week (n, %)			**<0.0001**			**<0.0001**
2 times	25 (14.5)	31 (33.7)		28 (14.5)	28 (38.9)	
3 times	148 (85.5)	61 (66.3)		165 (85.5)	44 (61.1)	
Length of dialysis session (hr) (mean±SD)	3.68 ± 0.38	3.7 ± 0.38	0.697	3.67 ± 0.38	3.7 ± 0.39	0.484
Urea mg/dL (mean±SD)	133.1 ± 41.1	136 ± 42.7	0.598	129.8 ± 39.2	145.8 ± 45.9	**0.005**
Creatinine mg/dL (mean±SD)	9.42 ± 3.1	9.02 ± 2.96	0.309	9.27 ± 2.89	9.28 ± 3.05	0.919

### Genotype and allele frequencies according to depressive and anxiety levels

**Fig 1:**
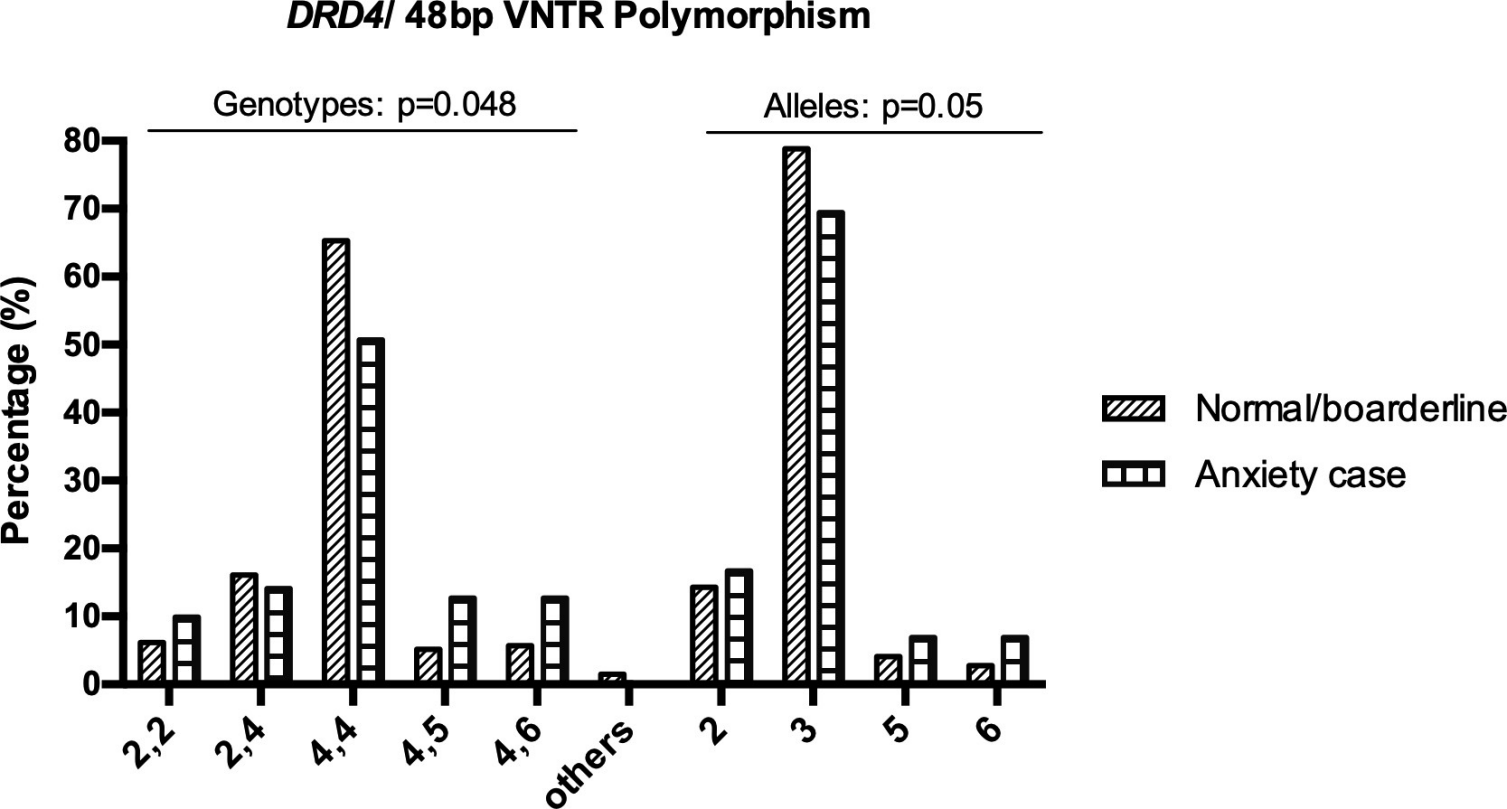
Genotype and allele frequencies of DRD4 (48 bp VNTR) polymorphism in controls and anxiety cases. Analysis was conducted using Chi-square or Fisher exact test when appropriate. The only genotype that remained significantly associated with anxiety at logistic regression is 4/5 genotype (Table 3).

Genotype and allele frequencies of the *DRD4* VNTR polymorphism were significantly different between anxiety cases versus normal-borderline subjects (Table 2). The frequency of the homozygous repeat 4,4 genotype was significantly lower in HADS-A cases (*p* = 0.023), while the 4,5 repeat was significantly higher (*p* = 0.04) in cases compared to the normal-borderline group. At the allelic level, a marginally significant difference was observed in the frequency of 5 and 6 alleles among HADS-A subgroups (*p* = 0.05). The frequency of the 4 allele was significantly lower in subjects with anxiety compared to normal subjects (69.4% vs. 78.8%, *p* = 0.025), while 6 allele frequency was higher in cases compared to subjects without borderline anxiety (6.9% vs. 2.8%, *p* = 0.032). However, the genotype and allele frequencies of the *DRD4* VNTR polymorphism did not differ by depression level (HADS-D). Fig 1 depicts the genotype and allele frequencies of DRD4 (48 bp VNTR) polymorphism in controls and anxiety cases, which is the only polymorphism that remained significant at the adjusted analysis as detailed below.

**Table 2:** Genotype and allele frequencies of DRD4 and SLC6A4 polymorphisms by depression and anxiety status.

	Depression (HADS-D)			Anxiety (HADS-A)		
Genotype/ Allele	Normal-borderline (173)	Case (92)	P	Normal-borderline (193)	Case (72)	P
SLC6A4 (5-HTTLPR) genotypes/alleles (n, %)						
LL	68 (39.3)	42 (45.6)	0.304	75 (38.9)	35 (48.6)	**0.038**
LS	57 (33)	22 (23.9)		66 (34.2)	13 (18.1)	
SS	48 (27.7)	28 (30.4)		52 (26.9)	24 (33.3)	
*L*	193 (55.8)	106 (57.6)	0.686	216 (56)	83 (57.6)	0.729
*S*	153 (44.2)	78 (42.4)		170 (44)	61 (42.3)	
rs25531 genotypes/alleles (n, %)						
AA	162 (93.6)	87 (94.6)	0.764	182 (94.3)	67 (93.1)	0.705
AG/GG	11 (6.4)	5 (5.4)		11 (5.7)	5 (6.9)	
A	334 (96.5)	178 (96.7)	0.9	374 (96.9)	138 (95.8)	0.55
G	12 (3.5)	6 (3.3)		12 (3.1)	6 (4.2)	
SLC6A4 (Tri-allelic-phased genotype) (n, %)						
LA/LA	54 (31.2)	23 (25)	0.057	59 (30.6)	18 (25)	**0.009**
LA/LG	5 (2.9)	3 (3.3)		5 (2.6)	3 (4.2)	
LA/SA	55 (31.8)	22 (23.9)		64 (33.2)	13 (18.1)	
SA/SA	43 (24.9)	26 (28.3)		47 (24.4)	22 (30.6)	
LA/superlong	10 (5.8)	16 (17.4)		12 (6.2)	14 (19.4)	
Others	6 (3.5)	2 (2.2)		6 (3.1)	2 (2.8)	
LA	179 (51.7)	87 (47.3)	0.072	200 (51.8)	66 (45.8)	**0.034**
LG	6 (1.7)	3 (1.63)		6 (1.5)	3 (2.1)	
SA	145 (41.9)	75 (40.8)		162 (42)	58 (40.3)	
SG	6 (1.7)	3 (1.6)		6 (1.5)	3 (2.1)	
Superlong	10 (2.9)	16 (8.7)		12 (3.2)	14 (9.7)	
DRD4-Exon III genotypes/alleles (n, %)						
2,2	11 (6.4)	8 (8.7)	0.147	12 (6.2)	7 (9.9)	**0.048**
2,4	30 (17.4)	11 (12)		31 (16.1)	10 (14.1)	
4,4	109 (63.4)	53 (57.6)		126 (65.3)	36 (50.7)	
4,5	10 (5.8)	9 (9.8)		10 (5.2)	9 (12.7)	
4,6	9 (5.2)	11 (12)		11 (5.7)	9 (12.7)	
Others	3 (1.7)	0 (0)		3 (1.5)	0 (0)	
2	52 (15)	27 (14.7)	0.389	55 (14.3)	24 (16.7)	**0.05**
4	267 (77.2)	137 (74.5)		304 (78.8)	100 (69.4)	
5	17 (4.9)	9 (4.9)		16 (4.1)	10 (6.9)	
6	10 (2.9)	11 (3.9)		11 (2.8)	10 (6.9)	

The genotype but not the allele frequency of 5-HTTLPR variants was different between patients, according to anxiety (HADS-A) scores (Table 2). For example, the proportion of the heterozygous LS genotype was 47% lower in cases compared to subjects without anxiety (18.1% vs. 34.2%, *p* = 0.011). Using the bi- and tri-allelic phased genotype of *SLC6A4* that comprises 5-HTTLPR and rs25531 variants, data show that the frequency of LA/SA was significantly lower, while LA/superlong was 2.1-fold higher in anxiety cases as compared to patients without borderline anxiety (18.1% vs. 33.2%, *p* = 0.016; 19.4% vs. 6.2%, *p* = 0.001, respectively). Also, the frequency of the S/superlong genotype was 2-fold higher in anxiety cases compared to those without anxiety (9.7% vs. 3.2%, *p* = 0.002). The genotype and allele frequencies of 5-HTTLPR and rs25531 were not different between patients stratified according to depression scores (HADS-D) (Table 2).

### Associations of DRD4 and SLC6A4 polymorphisms with depression and anxiety using adjusted analysis

The association of *DRD4* and *SLC6A4* genetic polymorphisms with anxiety and depression in HD patients was also assessed after adjusting for demographic and clinical covariates. As listed in Table 3, carriers of the 4,5 genotype of the *DRD4* VNTR polymorphism had 4.25 odds (*p* = 0.028) of developing anxiety symptoms compared to the carriers of the wild type 2,4 genotype, while *DRD4* VNTR alleles were not linked to anxiety. The genotypes and alleles of the 5-HTTLPR and rs25531 polymorphisms were not associated with anxiety in HD patients (*p* > 0.05). Furthermore, none of the studied polymorphisms were associated with depression after adjusting for other variables (Table 4). Some of the demographic and clinical factors were also significant predictors of anxiety or depression, such as education, income, and number of dialysis sessions per week.

**Table 3:** The effect of DRD4 and SLC6A4 polymorphisms on anxiety adjusting for other covariates.

Model	Covariate	Odds ratio	Confidence interval (95%)	P
5-HTTLPR Genotype	Education/ Illiterate	-	-	-
	Education/ Junior school	0.46	0.21–1.02	0.057
	Education/ High school	0.27	0.12–0.59	0.001
	Education/ College or over	0.27	0.11–0.63	0.003
	Two dialysis session/week	-	-	-
	Three dialysis session/week	0.39	0.20–0.75	0.005
	5-HTTLPR/ SS	-	-	-
	5-HTTLPR/ LS	0.74	0.35–1.55	0.426
	5-HTTLPR/ LL	1.13	0.59–2.16	0.722
5-HTTLPR Allele	Education/ Illiterate	-	-	-
	Education/ Junior school	0.41	0.22–0.75	0.004
	Education/ High school	0.34	0.18–0.61	<0.0001
	Education/ College or over	0.21	0.10–0.41	<0.0001
	Diabetes	1.6	1.04–2.48	0.034
	Income <250 JD	-	-	-
	Income 250–500 JD	2.06	1.26–3.36	0.004
	Income >500 JD	1.53	0.72–3.25	0.27
	Two dialysis session/week	-	-	-
	Three dialysis session/week	0.39	0.24–0.66	<0.0001
	Urea Concentration	1.01	1.0–1.01	0.018
	*S* allele	-	-	-
	*L* allele	1.09	0.71–1.69	0.68
rs25531 Genotype	Education/ Illiterate	-	-	-
	Education/ Junior school	0.4	0.17–0.96	0.041
	Education/ High school	0.33	0.14–0.78	0.011
	Education/ College or over	0.2	0.07–0.54	0.002
	Income <250 JD	-	-	-
	Income 250–500 JD	2.07	1.03–4.12	0.04
	Income >500 JD	1.55	0.53–4.52	0.423
	Two dialysis session/week	-	-	-
	Three dialysis session/week	1.55	0.53–4.52	0.012
	rs25531 AG/GG	-	-	-
	rs25531 AA	0.75	0.22–2.55	0.648
rs25531 Allele	Education/ Illiterate	-	-	-
	Education/ Junior school	0.41	0.22–0.75	0.004
	Education/ High school	0.33	0.18–0.61	<0.0001
	Education/ College or over	0.2	0.09–0.41	<0.0001
	Diabetes	1.6	1.04–2.48	0.034
	Income <250 JD	-	-	-
	Income 250–500 JD	2.07	1.27–3.38	0.004
	Income >500 JD	1.55	0.73–3.29	0.259
	Two dialysis session/week	-	-	-
	Three dialysis session/week	0.41	0.25–0.67	<0.0001
	Urea Concentration	1	1.00–1.01	0.017
	G allele	-	-	-
	A allele	0.66	0.22–1.99	0.457
DRD4-Exon III Genotype	Education/ Illiterate	-	-	-
	Education/ Junior school	0.4	0.17–0.97	0.042
	Education/ High school	0.35	0.15–0.83	0.017
	Education/ College or over	0.19	0.07–0.54	0.002
	Income <250 JD	-	-	-
	Income 250–500 JD	2.08	1.02–4.23	0.043
	Income >500 JD	1.44	0.48–4.34	0.516
	Two dialysis session/week	-	-	-
	Three dialysis session/week	0.48	0.21–0.89	0.024
	DRD4/ (2,4)	-	-	-
	DRD4/ (2,2)	1.83	0.46–7.25	0.389
	DRD4/ (4,4)	1.03	0.41–2.58	0.947
	DRD4/ (4,5)	4.25	1.17–15.5	**0.028**
	DRD4/ (4,6)	2.47	0.69–8.77	0.162
DRD4-Exon III Allele	Education/ Illiterate	-	-	-
	Education/ Junior school	0.41	0.16–1.38	0.004
	Education/ High school	0.33	0.16–1.08	<0.0001
	Education/ College or over	0.2	0.31–0.83	<0.0001
	Diabetes	1.71	1.09–2.67	0.018
	Income <250 JD	-	-	-
	Income 250–500 JD	2.08	1.27–3.41	0.004
	Income >500 JD	1.52	0.71–3.26	0.284
	Two dialysis session/week	-	-	-
	Three dialysis session/week	0.41	0.25–0.67	<0.0001
	Urea Concentration	1.01	1.001–1.002	0.017
	DRD4/ 2	0.47	0.16–1.38	0.168
	DRD4/ 4	0.42	0.16–1.08	0.071
	DRD4/ 5	1.09	0.31–3.83	0.888
	DRD4/ 6	-	-	-

**Table 4:** The effect of DRD4 and SLC6A4 polymorphisms on depression adjusting for other covariates.

Model	Covariate	Odds ratio	Confidence interval (95%)	P
5-HTTLPR Genotype	Education/ Illiterate	-	-	-
	Education/ Junior school	0.46	0.21–1.02	0.056
	Education/ High school	0.27	0.12–0.59	0.001
	Education/ College or over	0.27	0.11–0.63	0.003
	Hypertension	1.83	0.96–3.5	0.066
	Two dialysis session/week	-	-	-
	Three dialysis session/week	0.39	0.2–0.75	0.005
	5-HTTLPR/ SS	-	-	-
	5-HTTLPR/ LS	0.74	0.35–1.55	0.426
	5-HTTLPR/ LL	1.13	0.59–2.16	0.722
5-HTTLPR Allele	Education/ Illiterate	-	-	-
	Education/ Junior school	0.54	0.29–0.98	0.042
	Education/ High school	0.29	0.16–0.53	<0.0001
	Education/ College or over	0.29	0.15–0.54	<0.0001
	Hypertension	1.8	1.12–2.89	0.016
	Two dialysis session/week	-	-	-
	Three dialysis session/week	0.36	0.22–0.59	<0.0001
	*S* allele	-	-	-
	*L* allele	1.2	0.8–1.8	0.376
rs25531 Genotype	Education/ Illiterate	-	-	-
	Education/ Junior school	0.48	0.22–1.06	0.068
	Education/ High school	0.28	0.13–0.60	0.001
	Education/ College or over	0.26	0.11–0.63	0.003
	Two dialysis session/week	-	-	-
	Three dialysis session/week	0.38	0.19–0.73	0.004
	rs25531 AG/GG	-	-	-
	rs25531 AA	1.08	0.34–3.48	0.895
rs25531 Allele	Education/ Illiterate	-	-	-
	Education/ Junior school	0.55	0.3–0.99	0.045
	Education/ High school	0.29	0.16–0.53	<0.0001
	Education/ College or over	0.29	0.15–0.55	<0.0001
	Hypertension	1.81	1.12–2.9	0.015
	Two dialysis session/week	-	-	-
	Three dialysis session/week	0.36	0.22–0.58	<0.0001
	G allele	-	-	-
	A allele	1.001	0.34–2.93	0.998
DRD4-Exon III Genotype	Education/ Illiterate	-	-	-
	Education/ Junior school	0.48	0.22–1.07	0.072
	Education/ High school	0.28	0.12–0.61	0.002
	Education/ College or over	0.25	0.10–0.60	0.002
	Two dialysis session/week	-	-	-
	Three dialysis session/week	0.42	0.22–0.82	0.011
	DRD4/ (2,4)	-	-	-
	DRD4/ (2,2)	1.41	0.38–5.24	0.61
	DRD4/ (4,4)	1.36	0.59–3.02	0.487
	DRD4/ (4,5)	2.45	0.74–8.09	0.141
	DRD4/ (4,6)	2.46	0.74–8.19	0.144
DRD4-Exon III Allele	Education/ Illiterate	-	-	-
	Education/ Junior school	0.54	0.29–0.98	0.042
	Education/ High school	0.29	0.16–0.53	<0.0001
	Education/ College or over	0.29	0.15–0.55	<0.0001
	Hypertension	1.76	1.09–2.84	0.021
	Two dialysis session/week	-	-	-
	Three dialysis session/week	0.37	0..23–0.60	<0.0001
	DRD4/ 2	0.59	0.2–1.76	0.352
	DRD4/ 4	0.63	0.24–1.61	0.333
	DRD4/ 5	0.75	0.21–2.66	0.661
	DRD4/ 6	-	-	-

## Discussion

Depression and anxiety are common symptoms in HD patients. To the best of our knowledge, this study is the first genetic research that has evaluated the potential association of *DRD4* (48 bp VNTR) and *SLC6A4* (5-HTTLPR and rs25531) genetic polymorphisms with psychiatric symptoms among dialysis patients. The study revealed significant variations in the distribution of various genotypes and alleles of *DRD4* VNTR and 5-HTTLPR polymorphisms according to the anxiety scores of patients. Of note, this is the first study to suggest that the presence of the 4,5 genotype of *DRD4* exon III polymorphism is associated with an increased risk of anxiety symptoms among HD patients. Nevertheless, none of the tested polymorphisms was linked to depressive symptoms. Some of the clinical and demographic factors can also help in predicting psychiatric symptoms in HD patients.

The current study revealed that *DRD4* VNTR 4/5 genotype was associated with anxiety status in Jordanian HD patients and, as a consequence, has significantly advanced the state of research in this area. The evolution of such symptoms might be affected by environmental, biological, or different genetic factors [10]. A previous analysis of 196 Japanese subjects showed a significant association between short alleles (2–4 repeats) within the *DRD4* gene and personality trait of neuroticism, such as anxiety [26]. These observations support the findings of the current study. The *DRD4* gene affects the activity of dopamine and is involved in many neurological processes, it shows polymorphism, and is one of the various genes studied in connection with psychiatric disorders, anxiety, and stress [27]. The present study represents an essential milestone in genetic strategies to increase knowledge of the mechanism behind anxiety symptoms in HD patients.

The presence of the 7-repeat allele of the *DRD4* VNTR polymorphism was shown to be associated with treatment outcomes in ADHD and larger methylphenidate dosing [28]. Additionally, carriers of the long version (i.e., 7–11 repeats) were more likely to report depression symptoms [29]. A number of studies have associated the ≥7R allele to what may be considered as less effective dopamine functioning at the genetic and molecular levels [30, 31], which may contribute to depression development. The present study sample, however, did not show individuals with 7-repeat allele which might explain in part the observed lack of association between *DRD4* VNTR polymorphism and depression in HD subjects. Leung et al. reported a significantly increased prevalence of the 2-repeat allele at the *DRD4* gene in Han Chinese patients, whereas none of the subjects had the *DRD4* 7-repeat allele [32]. Of note, other studies reported a negligible association between the *DRD4* gene polymorphism and depression symptoms [33], which is in agreement with our findings.

The human serotonin transporter is a monoamine transporter protein, encoded by a single gene (*SLC6A4*) found on the long arm of chromosome 17 (17q11.2). This protein consists of 13–14 exons, spanning almost 35 kb. It constitutes 12–13 membrane-spanning domains [34]. The current study showed that the genetic variants (5-HTTLPR and rs25531) within the *SLC6A4* gene were not associated with psychiatric symptoms of anxiety and depression among HD patients after adjusting for other covariates. Our outcomes are in agreement with those of another study that documented negligible effects of the 5-HTTLPR polymorphism in the pathogenesis of depression among HD patients in Taiwan [10]. Previously, the 5-HTTLPR polymorphism had long been suggested to have a possible role in the pathogenesis of depression symptoms [19]. Also, there is evidence that people with the short (“*S*”) allele have lower serotonin transporter expression than long (“*L*”) allele carriers, and this could increase the risk of developing depression [35]. Nevertheless, meta-analyses and modern research studies with large sample size did not support the hypothesis of the interaction impact of the 5-HTTLPR genotype within the *SLC6A4* gene in the evolution of depression symptoms [36–38]. Additionally, data concerning the role of 5-HTTLPR polymorphism in the development of anxiety disorders are conflicting [39]. Perhaps, the risk of psychiatric distress in HD patients may be largely modulated by non-genetic factors or epigenetic alterations such as methylation to the promoter region of *SLC6A4* which resulted in decreased serotonin synthesis [40, 41]. Collectively, the role of 5-HTTLPR polymorphisms in the etiology of psychiatric symptoms requires further considerations.

Recent studies have focused on the analysis of genetic interaction between VNTRs in the same gene and its relation with psychiatric traits and personality. For example, the genetic interaction between HTTLPR and STin2 VNTRs within the *SLC6A4* gene in regulation of nicotine dependence was evaluated [42]. The results showed that carriers of S/S HTTLPR genotype showed a stronger association between STin2 10/10 variant and number of cigarettes smoke per day [42]. Another genetic investigation showed that the interaction between uVNTR and dVNTR in the monoamine oxidase A (MAOA) gene was significantly associated with nicotine dependence [43]. As the interaction between VNTRs within the gene may regulate its transcriptional expression, further studies are needed to estimate the role of genetic interaction between the DRD4 Exon III or 5-HTTLPR VNTR polymorphisms and other genetic markers in individual phenotype and the relation with psychiatric symptoms in HD patients.

This study has some limitations. Patient groups were not fully matched. Nevertheless, we did account for such discrepancies by the regression analysis method. Additional factors such as comorbidity index, which has not been considered in this research, might have affected the risk of psychological symptoms. It is important to estimate the integration of the special environmental and clinical factors with the linked genetic factors, such as the tryptophan hydroxylase and serotonin receptor that plays a crucial role in serotonin availability, levels, and function. Overall, the outcomes of our study provide additional clinical knowledge that could be applied in building a new molecular-genetic approach that seeks to minimize the severity of HD. This study should also animate more genetic and molecular examinations in various Arab populations to better understand the population’s genetic background and the psychopathology of HD, as well as to enhance the treatment plans.

In conclusion, the heterozygous genotype (4/5) in the *DRD4* gene could be a genetic risk factor linked with anxiety in HD. In future work, we suggest focusing on various candidate genes with further specific genetic polymorphisms and studying the epigenetic alterations for these genes at the genetic level. Different genetic studies including genetic haplotypes analysis will be also taken into consideration in further investigations to understand the effects of these genetic polymorphisms on individual phenotype. Additionally, as the research was carried out in six main dialysis centers in Jordan and the sample represents, originally, the north/middle of Jordan, another study on a more national scale might be justified.

## Supporting information

S1 File (DOCX)
