# Supplementary material for: Association of DRD4 exon III and 5-HTTLPR VNTR genetic polymorphisms with psychiatric symptoms in hemodialysis patients
Source: PLoS One. 2021 Mar 30;16(3):e0249284. doi: 10.1371/journal.pone.0249284 (PMC8009383; doi:10.1371/journal.pone.0249284)
Supplement: S1 File — (DOCX) [file pone.0249284.s001.docx]

**Appendix 1: Data collection sheet**

**Participant’s demographics and medical characteristics:**

1. Age (years): ………………
2. Gender: 1. Male 2. Female
3. Marital status: 1. Single 2. Married 3. Divorced 4. Widowed
4. Education level: 1. Illiterate 2. Junior School 3. High school 4. College or over
5. Height: _________Cm Weight: _________Kg calculated BMI: …………
6. Comorbidities: 1. Hypertension 2. Diabetes 3. None 4. Others ………………
7. Smoking status: 1. Yes 2. No
8. Income per month: <250 JD 250-500 JD >500 JD
9. Current Medications:
   .................................... .................................. ................................
   .................................... .................................. ................................
   .................................... .................................. ................................
10. Time since the first dialysis session: ..........................

Length of dialysis session (hours): ……………….

Number of dialysis sessions per week: …………

Dialysis characteristics: …………………………………………………………….

1. Serum Creatinine: ……………………
2. Serum urea: ……………..

**Appendix 2:**

Hospital Anxiety and Depression Scale (HADS) was used to assess anxiety or depression symptoms as published elsewhere (PMID: 6880820)

**The total score was categorized as follows:**

0-7: normal - border line case of anxiety or depression

>= 11-21: abnormal case (anxiety or depression)

**Appendix 3:**

Genotype analysis of *SLC6A4* (5-HTTLPR VNTR and rs25531) and *DRD4* (48 bp VNTR) polymorphisms:


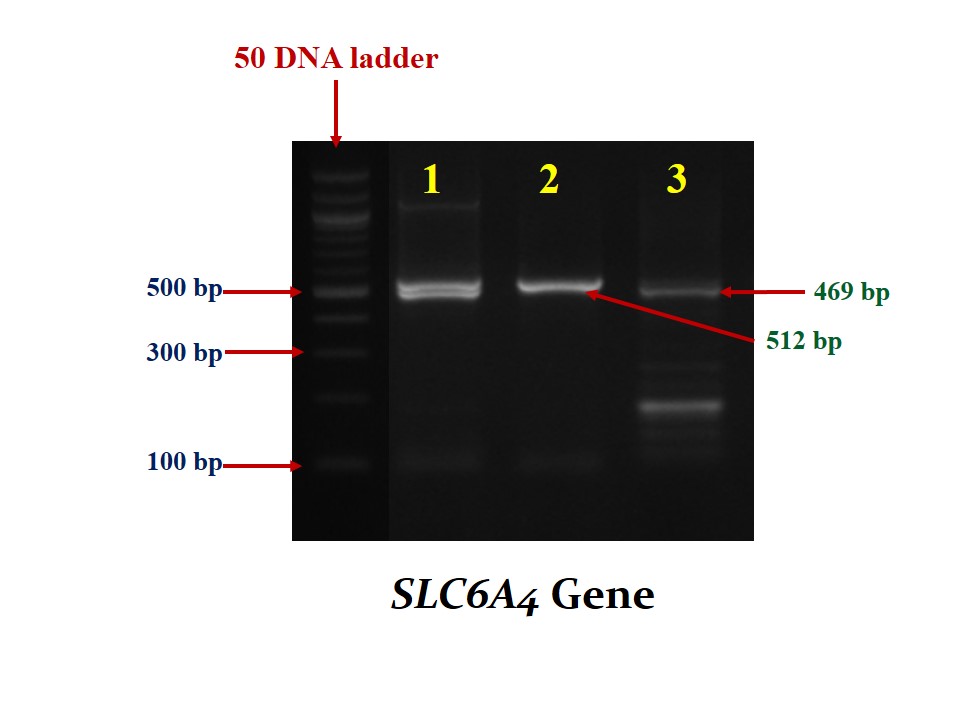


**Supplementary Figure 1.** Agarose gel electrophoresis of amplified genomic DNA for the 5-HTT (LL/LS/SS) in the *SLC6A4* gene. The larger PCR product size for 5-HTT (LL) referred to as (‘L’ allele) and the smaller size (SS) referred to (‘S’ allele). For example, in the panel representing the amplification products for 5-HTT, individuals who are homozygous for the smaller ‘S’ allele (469 base-pair) product is shown in lane 3 (genotype: SS) and the larger ‘L’ allele (512 base-pair) product is shown in lane 2 (genotype: LL). An individual with the heterozygous genotype (LS) is shown in lane 1.


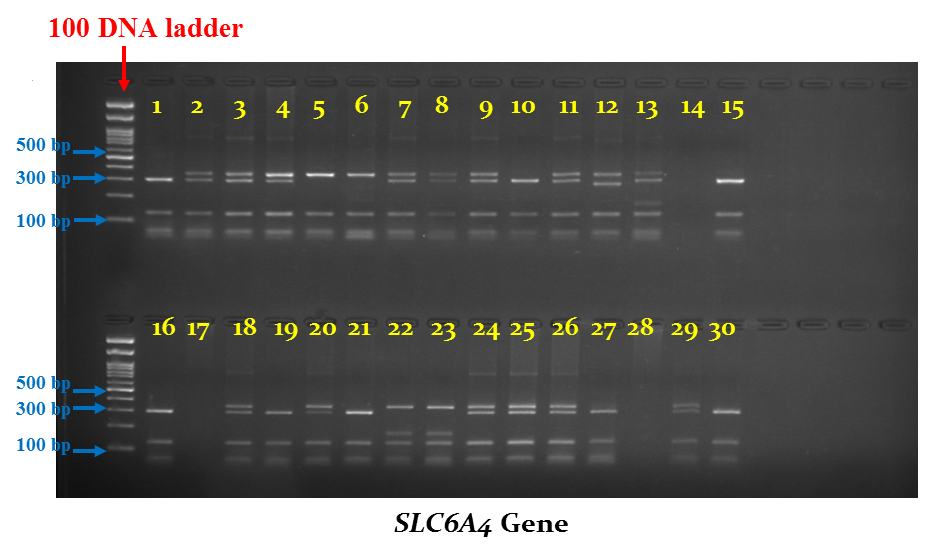


**Supplementary Figure 2.** (RFLP-PCR) ‘Triallelic approach’ assays were designed to genotype the rs25531 (A/G) in the presence of 5-HTT variants from 30 individuals. The genotype possibilities with presence of A/G SNP are shown by subdividing the 5-HTT alleles into *S_A_, S_G_, L_A_, L_G_* and Superlong. Genotypes thus are *L_A_/S_A_*: (2), (3), (4), (7), (8), (9), (11), and (12)*;* *L_A_/L_G_:* (22) and (23); *L_A_/L_A_*: (5) and (6); *S_A_/S_A_*: (1), (10) and (15)*;* *S_A_/S_G_*: (13)*;* *L_A_*/Superlong: (24), (25), and (26).


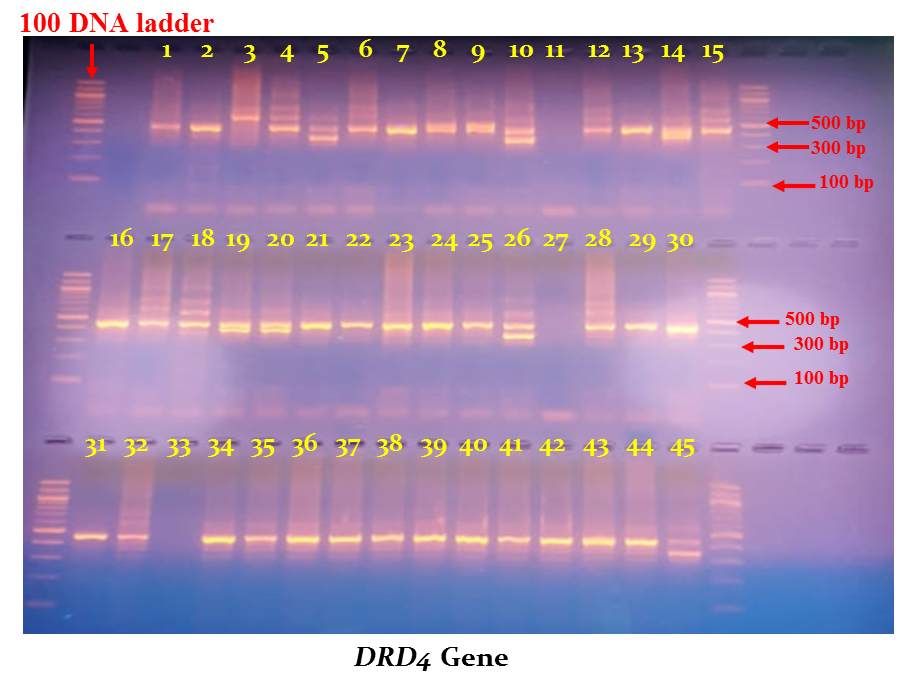


**Supplementary Figure 3.** PCR analysis of the dopamine receptor 4 gene (*DRD4*) from 45 individuals. Genotypes for this gene are: lane (14): 2/2 repeat type. Lane (5), (10), (26), and (45): 2/4 repeat type. Lanes (1), (2), (6), (7), (13), (16), (21-25), and (34-44): represent 4/4 repeat type. Lanes (4) and (15): represent 4/5 repeat type. Lanes (17) and (18): represent 4/6 repeat type.
